# The Distance Between the Cranial Nerve IX-X Root Entry/Exit Zone and the Pontomedullary Sulcus: MR Imaging Study in Patients With Hemifacial Spasm

**DOI:** 10.3389/fneur.2022.819488

**Published:** 2022-02-21

**Authors:** Jixia Fang, Gaoquan Lv, Dongliang Wang, Ruen Liu

**Affiliations:** ^1^Department of Neurosurgery, Peking University People's Hospital, Beijing, China; ^2^Department of Radiology, Peking University People's Hospital, Beijing, China

**Keywords:** the pontomedullary sulcus, MRI, hemifacial spasm (HFS), anatomic description, cranial nerve IX-X, root entry/exit zone

## Abstract

**Subject:**

To quantitatively describe the distance between the cranial nerve (CN) IX-X root entry/exit zone (REZ) and the pontomedullary sulcus in patients with hemifacial spasm (HFS).

**Methods:**

A total of 215 outpatients with HFS were recruited. Finally, 108 patients who yielded high-quality images were enrolled in the study. MRIs were reconstructed to measure the distance between the bilateral CN IX-X REZs and the corresponding pontomedullary sulcus.

**Results:**

Among the 108 patients, the ratio of males to females was 39/69, and the mean age was 57.9 ± 6.5 years. The ratio of left to right HFS involvement was 47/61. The average height was 1.62 ± 0.07 m, and the average body mass index (BMI) was 24.65 ± 2.97 kg/m^2^. The distance between the cephalic end of the CN IX-X REZ and the pontomedullary sulcus was 2.7 ± 0.9 mm. The distance between the caudal end of the CN IX-X REZ and the pontomedullary sulcus was 7.6 ± 1.1 mm. No monotonic relationship was found between distance and height or BMI in the scatter diagrams.

**Conclusions:**

The CN IX-X REZ is closely related to the pontomedullary sulcus in patients with HFS, and there is no difference between the left and right sides. The distances were not correlated with height or BMI in patients with HFS.

## Introduction

The glossopharyngeal nerve (cranial nerve [CN] IX) and the vagus nerve (CN X) enter/exit the brainstem at the rostral ventrolateral medulla, run through the prepontine cistern, and enter/exit the cranial cavity through the jugular foramen (1–3). The CN IX-X root entry/exit zone (REZ) can be identified through the retrosigmoid approach at the beginning, and because there is little anatomical variation in its position, it serves as a good anatomical marker.

According to intraoperative experience, the rostral end of the CN IX-X REZ is separated from CN VII-VIII by 3–5 mm, while the caudal end is mostly within 10 mm. To our knowledge, specific data regarding the location of the CN IX-X REZ have not been previously reported. Therefore, we measured the distance between the pontomedullary sulcus and the CN IX-X REZ on MRI to provide a quantitative description of the CN IX-X REZ.

## Materials and Methods

All subjects in this study were recruited from the neurosurgery clinic of Peking University People's Hospital from February 2021 to April 2021. The subjects were experiencing hemifacial spasm (HFS) and were scheduled to undergo imaging examination to assist in diagnosis and differential diagnosis.

Patients were enrolled in the study according to the following inclusion criteria: (1) age over 18 years; and (2) complete head examination using a 3.0 T MRI system. The exclusion criteria were as follows: (1) inability to undergo 3.0 T MRI (due to claustrophobia, metal implants, etc.), or lack of high-quality imaging results; (2) posterior fossa malformation; (3) space-occupying lesions at the cerebellopontine angle (CPA); (4) previous surgical history of the CPA; and (5) refusal to participate in any study.

Patients signed an informed consent which informed that their MRI scan and data would be used in the study. The study was approved by the ethics committee of Peking University People's Hospital.

MRI examinations were performed using a 3.0 T system (PHILIPS Ingenia, Eindhoven, the Netherlands) with a head coil. After acquiring initial localization images including the whole posterior fossa, a T2-weighted (T2W) sequence (repetition time [TR] 2,000 ms, echo time [TE] 235 ms, field of view 150 ^*^150 mm, 200 ^*^ 0.6-mm-thick slices, gap −0.3 mm) was performed on an oblique axial plane perpendicular to the axis of the brain stem.

**Figure 1:**
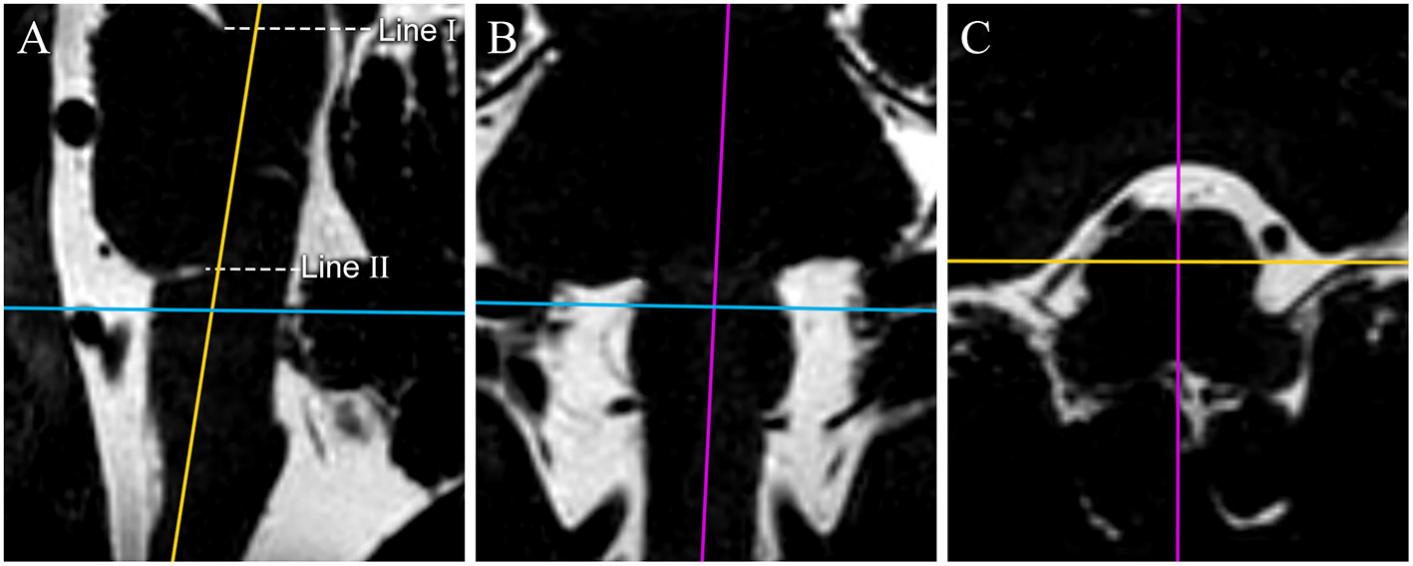
The reconstruction process of original MR image data. 3D-T2-weighted images are reconstructed in the sagittal plane **(A)**, coronal plane **(B)**, and transverse plane **(C)**. Color lines represent corresponding planes (purple: sagittal plane, yellow: coronal plane, and blue: transverse plane). 3D-T2-weighted images are reconstructed to get the median sagittal plane **(A)** according the anterior median sulcus and posterior median fissure of the medulla oblongata in the transverse plane image (the purple line on **C**) and the long axis of the medulla oblongata on the coronal plane image (the purple line on **B**). Line I passes through the superior pontine notch and the inferior edge of the quadrigeminal plate. Line II runs parallel to line I, passes through the inferior pontine notch, and is considered the pontomedullary sulcus. We made a hypothesis in the study: through our adjustment of the plane, the pontomedullary sulcus we confirmed by line II could be on the same horizontal plane. The transverse images are reconstructed based on the auxiliary line on the median sagittal plane image for surveying the CN IX-X REZ.

All the sequences were transferred to a workstation (Philips IntelliSpace Portal) and then reconstructed and adjusted in sagittal plane, coronal plane and transverse plane images (0.3 mm thick slice) according to easily distinguishable anatomical markers on the medulla oblongata with the Multiple Plane Reformation (MPR) tool (Figure 1).

The line tool was used to make two auxiliary lines on the median sagittal images. The first line (I) passed through the superior pontine notch and the inferior edge of the quadrigeminal plate. The axial images were then adjusted to be parallel to the auxiliary line. The second line (II), running parallel to line I, passed through the inferior pontine notch, which was considered the pontomedullary sulcus (4) (Figure 1A).

**Figure 2:**
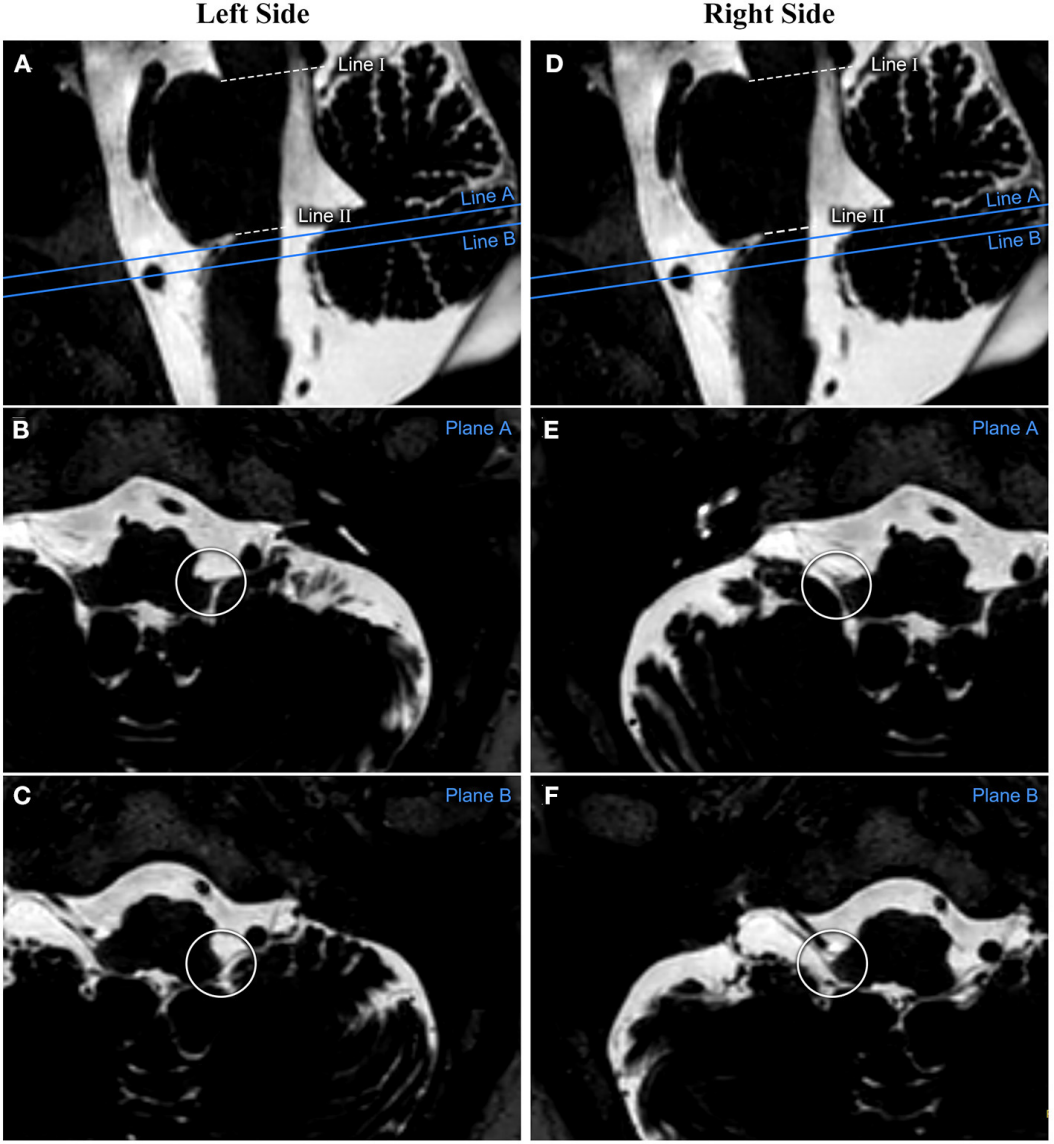
The measurement process performed on one patient's reconstructed image. The relationship between CN IX-X and the medulla oblongata is assessed based on the reconstructed transverse plane images. The cephalic and caudal planes of the CN IX-X REZ are marked on the sagittal image as planes **(A,B)**, respectively. **(A–C)** show the measurements from the patient's left side. **(D–F)** show the measurements from the patient's right side. CN, cranial nerve; REZ, root entry/exit zone.

All transverse plane images in which CN IX-X enters/exits the medulla oblongata were identified and marked on the median sagittal plane images. On the transverse plane images, the closest and farthest slices to the pontomedullary sulcus were recorded as slice A and slice B, respectively, and were indicated as line A and line B on the median sagittal plane images. The distance between line II and line A and between line II and line B was measured on the median sagittal plane images. These two values were considered the distances between the rostral and caudal ends of the CN IX-X REZ, respectively, and the pontomedullary sulcus. The distances for the REZs on the left and right sides were measured separately (Figure 2)

The data were analyzed using SPSS, version 22.0 (IBM, United States). Age, body mass index (BMI), height and distance are described by the mean and standard deviation. The paired sample *t*-test was used to evaluate whether there was a significant difference in the distance between the CN IX-X REZ and the pontomedullary sulcus on the left and right. Spearman correlation was used to assess the relationship between the distance and height and BMI.

## Results

**Figure 3:**
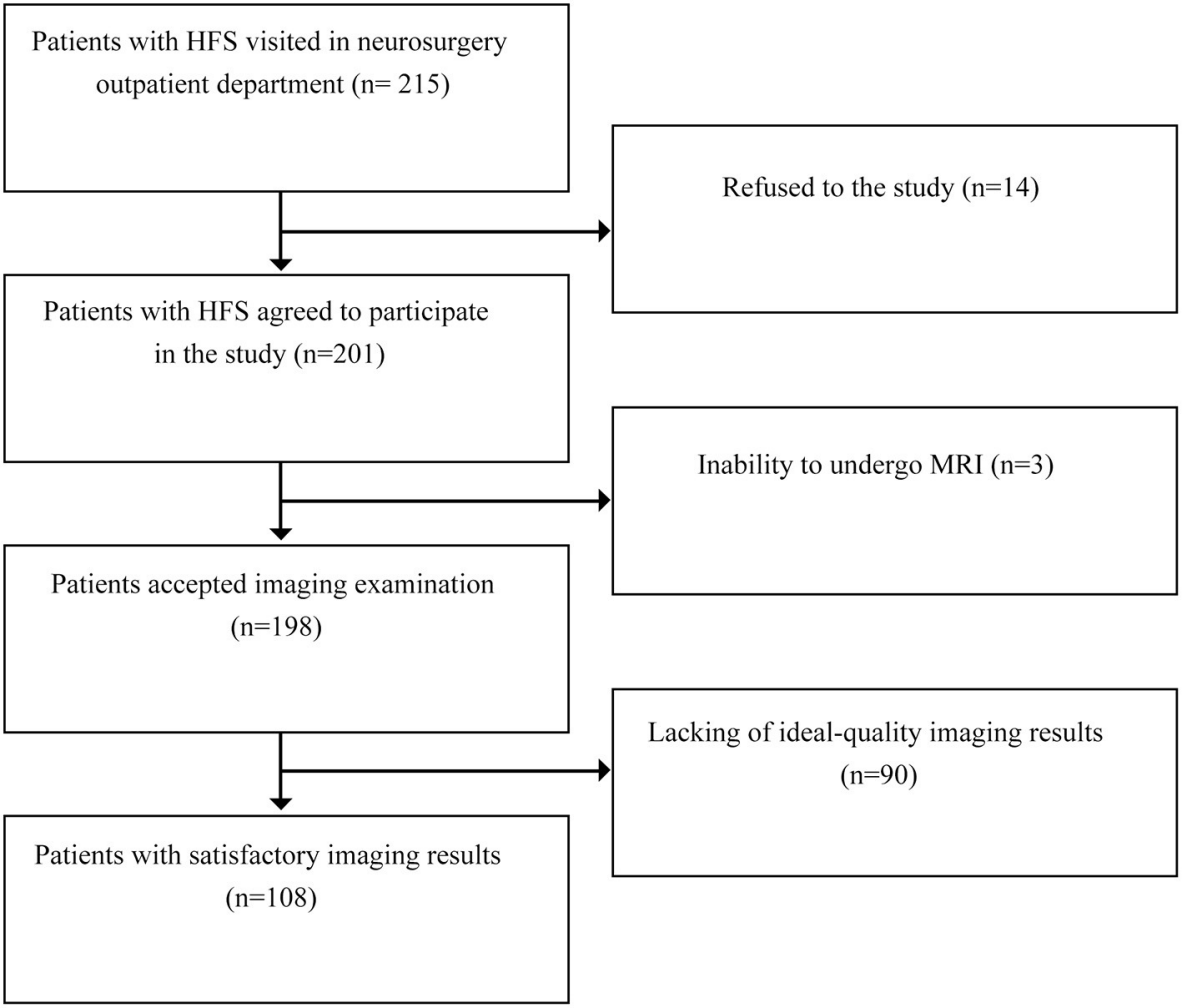
Flow diagram of literature search and study selection.

From February 2021 to April 2021, 215 patients with HFS visited the neurosurgical clinics, 198 of whom successfully underwent 3.0 T MRI. Ninety subjects were excluded for poor image quality, including 20 with poor images on both sides, 41 with poor images on the left and 29 with poor images on the right. Finally, 108 patients with HFS were enrolled in the study (Figure 3). The ratio of males to females was 39/69, and the mean age was 57.9 ± 6.5 years. The ratio of left to right HFS involvement was 47/61. The average height was 1.62 ± 0.07 m, and the average BMI was 24.65 ± 2.97 kg/m^2^ (Table 1).

**Table 1:** Demographic and study data of patients with HFS enrolled in the study.

**Variables**		
No. of cohort	108	
Sex ratio (M/F)	39/69	
Age (year)	57.9 ± 6.5	
BMI (kg/m^2^)	24.65 ± 2.97	
Left/Right affected	47/61	
Rostral (mm)^*^		*P*
Left side	2.6 ± 0.9	0.520
Right side	2.8 ± 0.9	
Mean	2.7 ± 0.9	
**Caudal (mm)^#^**		*P*
Left side	7.5 ± 1.1	0.681
Right side	7.7 ± 1.2	
Mean	7.6 ± 1.1	

* *The distances between the rostral ends of the CN IX-X Root Entry/Exit Zone and the Pontomedullary Sulcus*.

# *The distances between the caudal ends of the CN IX-X Root Entry/Exit Zone and the Pontomedullary Sulcus*.

The distances between the cephalic end of the CN IX-X REZ and the pontomedullary sulcus were 2.8 ± 0.9 mm on the right and 2.6 ± 0.9 mm on the left (*P* = 0.520); the mean distance was 2.7 ± 0.9 mm. The distances between the caudal end of the CN IX-X REZ and the pontomedullary sulcus were 7.7 ± 1.2 mm on the right and 7.5 ± 1.1 mm on the left (*P* = 0.681); the mean distance was 7.6 ± 1.1 mm (Table 1).

**Figure 4:**
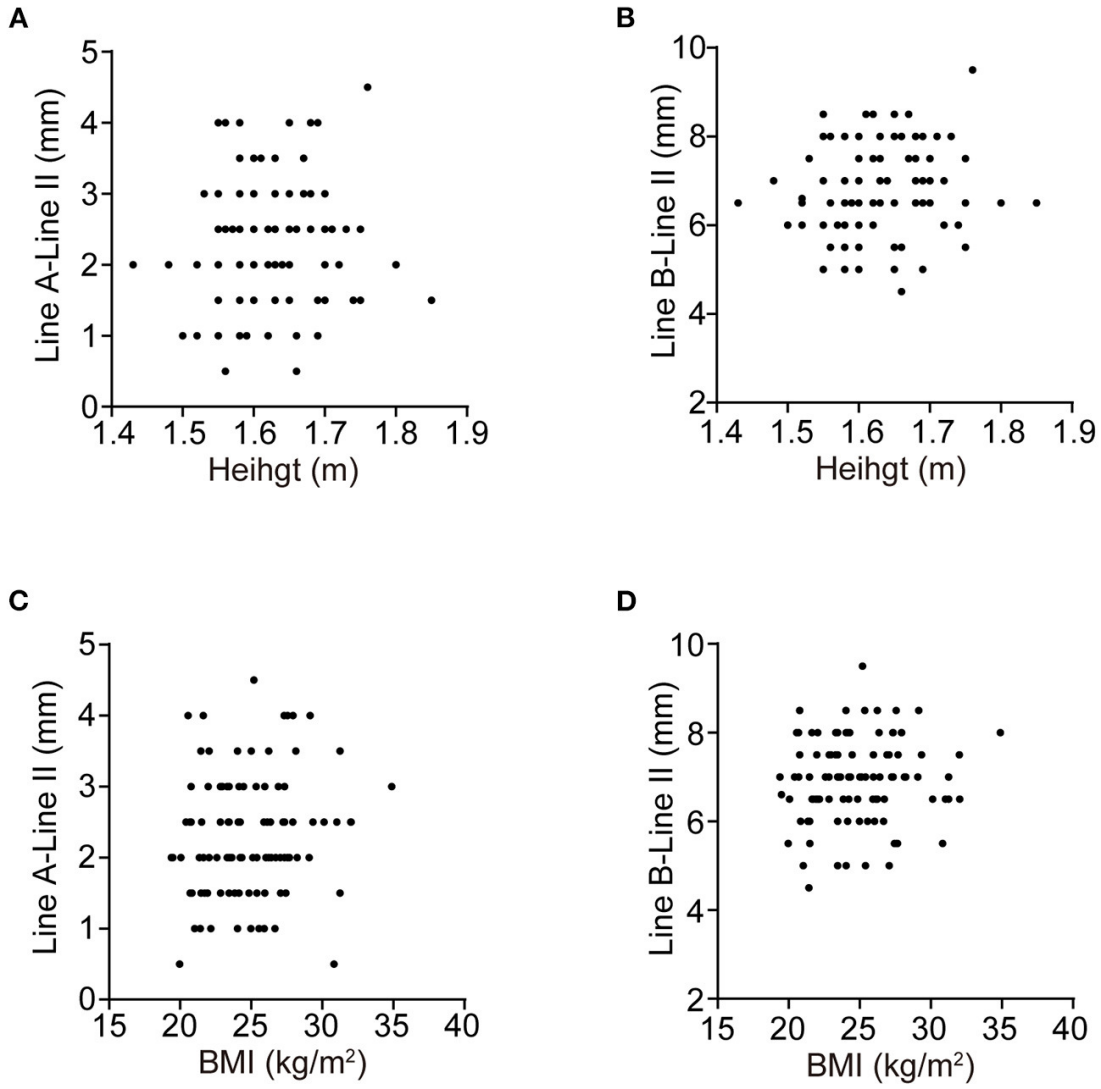
The scatter diagrams. **(A–D)** are the scatter diagrams of the distances between the cephalic, caudal end of the CN IX-X REZ, and the pontomedullary sulcus and the height, BMI, respectively. No monotonic relationship is found.

No monotonic relationship was found in the scatter diagrams between distance and either height or weight (Figure 4). Spearman correlation analysis was conducted for the distance between the cephalic end of the CN IX-X REZ and the pontomedullary sulcus and height (*r* = 0.086, *P* = 0.640) and BMI (*r* = 0.214, *P* = 0.240), and for the distance between the caudal end of the REZ and the pontomedullary sulcus and height (*r* = 0.095, *P* = 0.562) and BMI (*r* = 0.195, *P* = 0.303).

## Discussion

The REZs of CN IX and CN X are adjacent and located at the rostral ventrolateral medulla (RVLM). Neural interconnections between CN IX and CN X can be observed in some individuals (5). After emerging from the postolivary sulcus, CN IX-X can be divided into three portions: cisternal, jugular foramen, and extracranial.

CN IX-X are both mixed cranial nerves, arise from the same nuclei and have similar functions (2, 5, 6). Stimulation of CN IX and of CN X produces comparable results in aborting seizures in an animal model (7). In a study of neurogenic hypertension, the CN IX-X REZ was regarded as the main research object (8).

**Figure 5:**
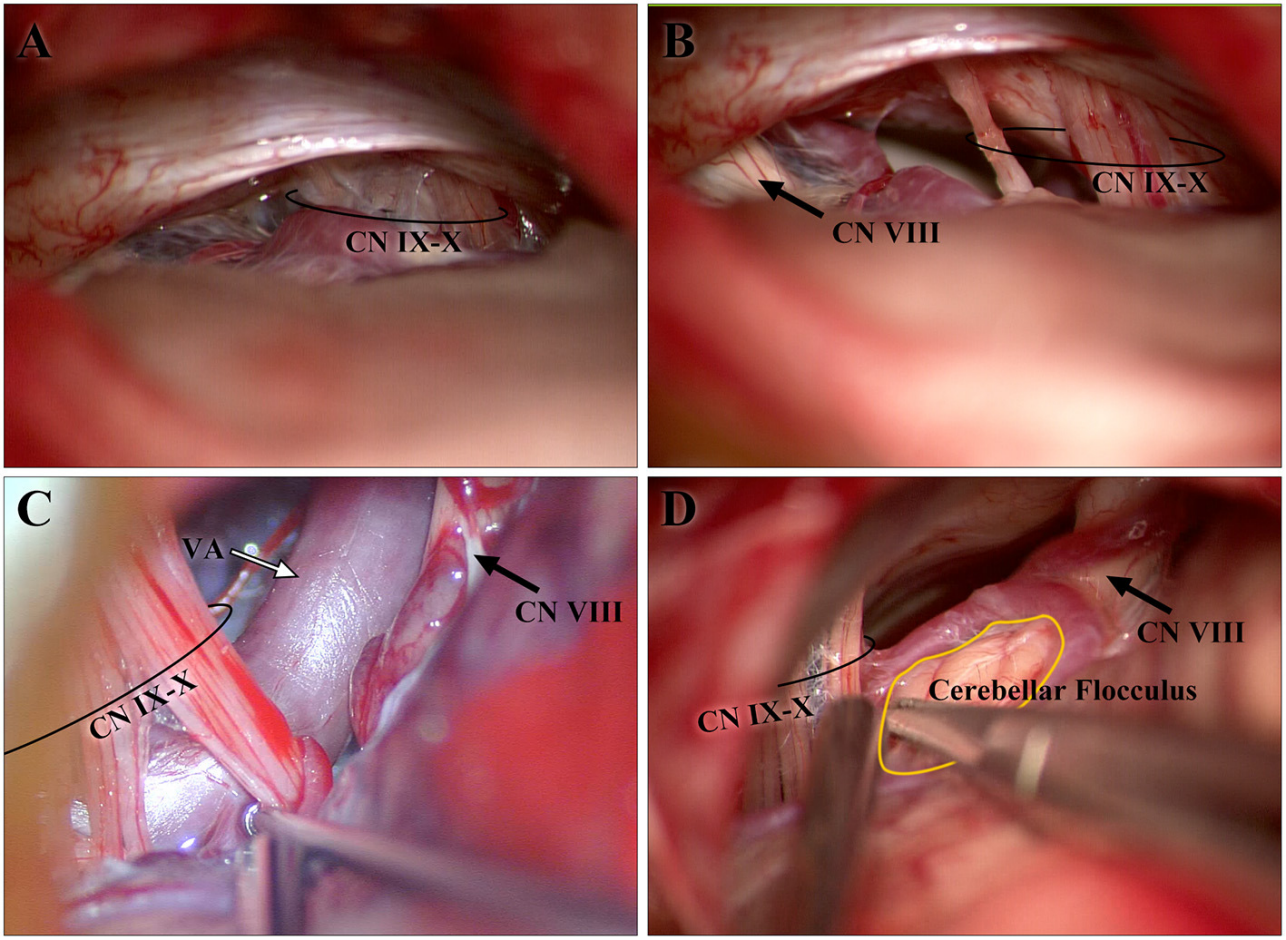
Images from intraoperative microscopy. **(A,B)** are both from one patient. The CN IX-X shown in **(A)** emerged after retraction of the cerebellum during the early stage of the operation, whereas CN VII-VIII had not yet become visible. The CN VII-VIII in **(B)** appeared after sharp separation and adjustment of the optical axis of the microscope. **(B)** The cisternal segments of CN IX-X and CN VII-VIII are shown. As CN IX-X and CN VII-VIII arise from the brainstem and gradually separate, the distance is longer than that in the study. **(C,D)** show the developed cerebellar flocculus and vessels around the CN IX-X REZ, respectively. CN, cranial nerve; REZ, root entry/exit zone.

When entering the cranium through the retrosigmoid approach, mainly used to perform microvascular decompression (MVD) for the treatment of HFS, CN IX-X is often the first cranial nerve that appears in the operation field (Figure 5A). According to the position of CN IX-X in the surgical field, we can determine whether the bone window is appropriate and locate the position of the REZ of CN VII-VIII before exposure of the CNs themselves, which helps protect them (Figure 5B).

However, published data rarely described the distance between the CN IX-X REZ and the CN VII-VIII REZ. Anatomic variations are mostly observed in the extracranial course of CN IX-X (9, 10). Fewer variants in the intracranial course have been reported to the author's knowledge, allowing an anatomical description of the CN IX-X REZ. On MR images, the signal intensity of the cranial nerve is consistent with that of the brainstem. After CN VII-VIII derives from the pontomedullary sulcus, it will travel along the pons for a short distance, called the transitional zone, and then enter the prepontine cistern (11). Therefore, on MR images, the distance between the REZ of CN VII-VIII and the REZ of CN IX-X is difficult to measure directly. The pontomedullary sulcus is an obvious, easily identified sulcus on the median sagittal plane and can be considered the CN VII-VIII REZ. CN IX-X enters/exits from the medulla oblongata in a nearly vertical direction, and so the CN IX-X REZ can be determined on the axial plane.

The brainstem is not completely symmetrical and is subject to rotation (12). The reconstructed MR image integrates the medulla oblongata into a coordinate system to reduce differences in measurement. Cerebellar flocculus or vessels can be seen in the CPA; along with tortuous vessels, they are the reasons for the poor-quality imaging of the CN IX-X REZ, for which dozens of subjects were excluded from the study (Figures 5C,D). Some individuals possess a cranial part of the accessory nerve (CN XI) (13), which may increase the distance between the caudal end of the CN IX-X REZ and the pontomedullary sulcus but cannot currently be determined on MR images. We designed specific steps to reconstruct the image, and the reconstructed image was adjusted according to easily identifiable anatomical markers to reduce the subjective differences in the measurements.

CN IX-X REZ is adjacent the cerebellomedullary fissure. Measurement based on the para-sagittal plane of the cerebellomedullary fissure could obtain the distance between the CN IX-X REZ and the nearest pontomedullary sulcus (14). These data can better reflect the anatomy in the individual. But in patients with HFS, responsible vessels such as vertebral arteries or other tortuous arteries at the RVLM and the ventrolateral pontine are adjacent to the pontomedullary sulcus and lead to unsatisfactory display of the lateral part of the pontomedullary sulcus (adjacent to the CN IX-X REZ) on the para-sagittal plane of the cerebellomedullary fissure. We made a hypothesis in the study: through our adjustment of the plane, the pontomedullary sulcus we confirmed by line II could be on the same horizontal plane, and the distance between the CN IX-X REZ and the pontomedullary sulcus would be equal to the vertical distance from this plane.

According to the study, the CN IX-X REZ and the pontomedullary sulcus were in close proximity, which is consistent with experience from surgery.

There is a close spatial relationship between the CN IX-X REZ and the pontomedullary sulcus (as well as with the REZ of CN VII-VIII). When performing MVD for HFS, if the CN IX-X REZ has been exposed in the surgical field but CN VIII still cannot be visualized, caution needs to be taken, as CN VII-VIII is within a few millimeters of the cephalic end of the CN IX-X REZ.

The pontomedullary sulcus shown in the para-sagittal plane and the pontomedullary sulcus shown in the median sagittal plane are not always on the same horizontal plane and might result in some errors in results. In normal people, using the classical method to measure the distance between the CN IX-X REZ and the pontomedullary sulcus would be helpful for our understanding of anatomy of CN IX-X.

The distance between the CN IX-X REZ and the pontomedullary sulcus was not correlated with height or BMI. Considering that we excluded 90 subjects with unsatisfactory image quality, which affected the results, more evidence is needed to support this conclusion.

We conducted the study in a cohort with HFS. Age, sex ratio, and BMI were different from those of healthy people. Obviously, the results cannot be generalized to this population.

In conclusion, the distance between the cephalic end of the CN IX-X REZ and the pontomedullary sulcus was 2.7 ± 0.9 mm, and the distance between the caudal end of the CN IX-X REZ and the pontomedullary sulcus was 7.6 ± 1.1 mm. The CN IX-X REZ is closely related to the pontomedullary sulcus in patients with HFS, with no difference between the left and right sides. Additionally, the distances are not correlated with height or BMI in patients with HFS.

## Data Availability Statement

The original contributions presented in the study are included in the article/supplementary material, further inquiries can be directed to the corresponding author/s.

## Ethics Statement

The studies involving human participants were reviewed and approved by Ethics Review Committee of Peking University People's Hospital. The patients/participants provided their written informed consent to participate in this study.

## Author Contributions

JF performed the experiment, and were major contributors in data collection, data analysis, and manuscript preparation. JF and GL reviewed all MRI images. DW provided intraoperative photos and edited the first draft. GL and DW were responsible for making the figures and tables. RL conceived and designed the study, and was a major contributor in critically revising the manuscript. All authors read and approved the final manuscript.

## Funding

Financial support was provided by Peking University People's Hospital (2017-T-01).

## Conflict of Interest

The authors declare that the research was conducted in the absence of any commercial or financial relationships that could be construed as a potential conflict of interest.

## Publisher's Note

All claims expressed in this article are solely those of the authors and do not necessarily represent those of their affiliated organizations, or those of the publisher, the editors and the reviewers. Any product that may be evaluated in this article, or claim that may be made by its manufacturer, is not guaranteed or endorsed by the publisher.
